# Variation of alkaloid contents and antimicrobial activities of *Papaver rhoeas* L. growing in Turkey and northern Cyprus

**DOI:** 10.1080/13880209.2017.1340964

**Published:** 2017-06-20

**Authors:** İlkcan Çoban, Gizem Gülsoy Toplan, Berna Özbek, Çağlayan Unsal Gürer, Günay Sarıyar

**Affiliations:** a Department of Pharmacognosy, Faculty of Pharmacy, Istanbul University, Istanbul, Turkey;; b Department of Pharmaceutical Microbiology, Faculty of Pharmacy, Istanbul University, Istanbul, Turkey;; c Faculty of Health Sciences, Cyprus International University, Haspolat, Lefkoşa, Cyprus

**Keywords:** alkaloids, antimicrobial activity

## Abstract

**Context:***Papaver rhoeas* L. (Papaveraceae) corn poppy, widely distributed in Turkey, is used to make a cough syrup for children, as a tea for disturbed sleep, for pain relief and as a sedative in folk medicine.

**Objective:** Samples of *P. rhoeas* collected from eight different locations in Turkey and three from northern Cyprus were investigated for their alkaloid content and screened for their antimicrobial activities.

**Materials and methods:** From the aerial parts of *P. rhoeas* samples, alkaloids were isolated by column and preparative thin-layer chromatography. The alkaloids were identified by comparing their spectral data (UV, IR and ^1^H-NMR) and TLC Rf values with those of authentic samples. The antimicrobial study was carried out by microbroth dilution technique against six strains of bacteria and three strains of fungi.

**Results:** Twelve different alkaloids belonging to proaporphine (mecambrine), aporphine (roemerine), promorphinan (salutaridine), protopine (coulteropine and protopine) and rhoeadine (epiglaucamine, glaucamine, glaudine, isorhoeadine, isorhoeagenine, rhoeadine and rhoeagenine) groups were isolated. The most significant activity was observed with the alkaloid extract of P8 against *Staphylococcus aureus* with a MIC value of 1.22 μg/mL and against *Candida albicans* with a MIC value of 2.4 μg/mL.

**Discussion:** The results indicate that *P. rhoeas* samples (P8 and P9), which contain roemerine as their major alkaloid, were the most active extracts.

## Introduction

*Papaver rhoeas* L. (Papaveraceae) – red poppy or corn poppy also named as ‘gelincik’ in Turkey – is an annual species of the section Rhoeadium Kadereit. The species is widely distributed in Turkey and used for the treatment of various diseases in folk medicine as a cough syrup for children, a tea for insomnia, a sedative and for pain relief. Fresh aerial parts are also used as food mainly in southwest of Turkey (Tuzlacı and Eryaşar Aymaz 2001; Koçyiğit and Özhatay 2004; Ecevit Genç and Özhatay 2006; Tuzlacı 2006). Poppy flowers are also used as a source of food colouring and for enhancing the flavour of herbal teas. Poppy flowers contain anthocyanin glycosides (cyanidin and mecocyanin), up to 12% isoquinoline alkaloids (50% is rhoeadine). They also contain mucilage and many ubiquitous substances (Nosalova et al. 2006). Table 1 summarizes folkloric uses of *P. rhoeas* in Turkey.

**Table 1.** Medicinal uses of *Papaver rhoeas* L. in folk medicine in Turkey.

Parts used	Uses	References
Petals	Sedative in children, blood sugar lowering	Yücel and Tülükoğlu (2000)
Flowers	Against hemorrhoids	Ecevit Genç and Özhatay (2006)
Young sprouts	Mild sedative	Tuzlacı and Emre Bulut (2007)
Roots	Antihelmintic	Tuzlacı (2006) and Tuzlacı and Erol (1999)
Petals	Against kidney stones	Aslan et al. (2007)
Flowers	Antipyretic	Tuzlacı (2006) and Tuzlacı and Sadıkoğlu (2007)
Flowers	Against asthma	Tuzlacı (2006) and Tuzlacı and Sadıkoğlu (2007)
Flowers	Insomnia	Tuzlacı (2006) and Tuzlacı and Sadıkoğlu (2007)
Petals	Antidiarrhoea	Tuzlacı (2006) and Tuzlacı and Sadıkoğlu (2007)
Flowers	Antitussive	Tuzlacı (2006) and Tuzlacı and Alparslan (2007)
Flowers	Antispasmodic	Tuzlacı (2006) and Tuzlacı and Alparslan (2007)
Flowers	Menstrual disorders	Tuzlacı (2006) and Tuzlacı and Alparslan (2007)
Aerial parts	Antirheumatism	Kültür (2007)
Petals	Sore throat	Kültür (2007)
Petals	Demulcent	Kültür (2007)
Petals	Immunstimulant	Kültür (2007)
Petals	Against nose bleeding	Kültür (2007)
Petals	Mouth wounds in children	Tuzlacı (2006) Tuzlacı and Eryaşar Aymaz (2001)
Leaves	Tonic	Tuzlacı (2006) and Tuzlacı and Eryaşar Aymaz (2001)
Leaves	Against jaundice	Tuzlacı (2006) and Tuzlacı and Eryaşar Aymaz (2001)
Petals	Galactagogue	Kültür (2007)

In our previous studies on the alkaloids of *P. rhoeas* of Turkish origin, we reported the chemotypes of this species containing rhoeadine, proaporphine and benzylisoquinoline types as major alkaloids (Kalav and Sarıyar 1989; Ünsal et al. 2007). One of the samples of corn poppy collected from southwest region of Turkey has been investigated for its antimicrobial activity using a microbroth dilution technique and diethyl ether, chloroform and acetone extracts of the aerial parts of the plant had showed a good activity against *Staphylococcus aureus* with a MIC value of 39.06 μg/mL (Ünsal et al. 2009). As a continuation of our work on this species, we now report the isolation of major alkaloids and antimicrobial activities of both methanol and tertiary alkaloid extracts of 11 samples.

## Materials and methods

Samples of *P. rhoeas* were collected from 11 different locations at the flowering stage. Collection data are given in Table 2. Voucher specimens have been identified by G. Sarıyar and deposited in the Herbarium of the Faculty of Pharmacy at Istanbul University (ISTE).

**Table 2.** Collection data of samples of *P. rhoeas* L. (P1–P11).

Sample no.	Location/date of collection	ISTE no.
P1	Uzunköprü, Edirne, Turkey/8 May 2008	85919
P2	Karabük, Turkey/18 May 2008	85923
P3	Eskişehir, Turkey/1 May 2008	85918
P4	Çine, Aydın, Turkey/16 May 2008	85921
P5	Bodrum, Muğla, Turkey/19 April 2008	84601
P6	Fethiye, Muğla, Turkey/20 May 2008	85917
P7	Malatya, Turkey/15 May 2008	85922
P8	Şanlıurfa, Turkey/15 May 2009	86164
P9	Girne, Northern Cyprus/12 May 2009	86050
P10	Bellapais, Girne, Northern Cyprus/April 2015	110445
P11	Lefkoşa, Magosa, Northern Cyprus/April 2015	110446

### Extraction and isolation of alkaloids

The dried aerial parts of *P. rhoeas* (P1–P11) samples were each percolated with methanol at room temperature and concentrated under vacuum. The residue was taken up in 5% hydrochloric acid. The acid extract was first washed with light petroleum and then with diethyl ether. The aqueous layer was made alkaline with NH_4_OH to pH 7–8 and extracted successively with CHCl_3_. The combined CHCl_3_ extracts were dried over anhydrous Na_2_SO_4_, filtered and concentrated under vacuum to yield the tertiary alkaloid extracts. Table 3 shows the amount of the plant material used for the extraction and tertiary alkaloid extracts obtained.

**Table 3.** The plant material used for the extraction and yield of the tertiary alkaloid extracts.

Sample no.	Material weight (g)	Yield of tertiaryalkaloid extracts (g) (%)
P1	722	0.68–0.09
P2	880	0.57–0.07
P3	647	0.75–0.12
P4	100	0.06–0.07
P5	824	1.24–0.15
P6	900	0.82–0.09
P7	780	0.76–0.1
P8	400	0.75–0.19
P9	955	1.60–0.17
P10	550	0.51–0.09
P11	700	1.29–0.18

The tertiary alkaloid extracts of P1–P11 were each separated on a column of silica gel (Kieselgel 60, 0.063–0.200 mm, 70–230 mesh) eluting with CHCl_3_ and CHCl_3_:MeOH (95:5, 90:10, 80:20). Fractions were evaporated and purified by preparative thin-layer chromatography on silica gel to afford the pure alkaloids. The solvent systems were cyclohexane:chloroform:diethylamine (7:2:1), cyclohexane:dietylamine (9:1) and (8:2). The identification of the alkaloids was carried out by comparing their spectral data (UV, IR, ^1^H-NMR and mass spectroscopy) and TLC Rf values with authentic samples. Nine different alkaloids belonging to aporphine (roemerine), protopine (coulteropine and protopine) and rhoeadine (epiglaucamine, glaudine, isorhoeadine, isorhoeagenine, rhoeadine and rhoeagenine) groups were isolated. Distribution of the alkaloids in the samples is shown in Table 4.

**Table 4.** Distribution of alkaloids in the samples of *P. rhoeas*(P1–P11).

Sample no.	Alkaloid type	Alkaloids
P1	Rhoeadine	Isorhoeagenine, rhoeagenine
P2	Rhoeadine	Isorhoeagenine, rhoeagenine
P3	Protopine, rhoeadine	Protopine, rhoeagenine
P4	Rhoeadine	Rhoeagenine
P5	Rhoeadine, protopine	Coulteropine, rhoeagenine
P6	Rhoeadine	Epiglaucamine, glaudine, isorhoeadine, isorhoeagenine, rhoeagenine
P7	Rhoeadine	Isorhoeagenine, rhoeadine, rhoeagenine
P8	Aporphine	Roemerine
P9	Aporphine	Roemerine
P10	Proaporphine, aporphine	Mecambrine, roemerine
P11	Promorphinan, rhoeadine	Salutaridine, glaucamine

### Antimicrobial activity tests

The antimicrobial activity tests were performed on methanol extracts obtained from the aerial parts of the samples (5 g) and on tertiary alkaloid extracts. Antimicrobial activity against *Staphylococcus aureus* ATCC 65538*, Staphylococcus epidermidis* ATCC 12228*, Escherichia coli* ATCC 11229, *Klebsiella pneumoniae* ATCC 4352, *Pseudomonas aeruginosa* ATCC 1539, *Proteus mirabilis* ATCC 14153, *Candida albicans* ATCC 10231, *Candida parapsilosis* ATCC 22019 and *Candida tropicalis* ATCC 750 were determined by the microbroth dilutions technique using the CLSI (2000, 2006) recommendations. Mueller–Hinton broth for bacteria, RPMI-1640 medium for yeast strain was used as the test media. The extracts were dissolved in dimethylsulphoxide (DMSO, 10 mg/mL) before the test for antimicrobial activity. Serial two-fold dilutions ranging from 5000 to 4.9 μg/mL were prepared in the medium. The inocula were prepared using a 4–6-h broth culture of each bacteria and 24 h culture of yeast strains adjusted to a turbidity equivalent to a 0.5 McFarland standard, diluted in broth media to give a final concentration of 5 × 10^5^ cfu/mL for bacteria and 0.5 × 10^3^ to 2.5 × 10^3^ cfu/mL for yeast in the test tray. The trays were covered and placed in plastic bags to prevent evaporation. The trays containing Mueller–Hinton broth were incubated at 35 °C for 18–20 h and the trays containing RPMI-1640 medium were incubated at 35 °C for 46–50 h.

The MIC was defined as the lowest concentration of compound giving complete inhibition of visible growth. All the micro-organisms were obtained from American Type Culture Collection (ATCC), Manassas, VA. Cefuroxime–Na and ceftazidime were used as a positive control for the tested bacteria, whereas clotrimazole was used as a positive control for yeast (CLSI 2000, 2006).

## Results and discussion

In this work, samples of *P. rhoeas* collected from eight different locations of Turkey and three from northern Cyprus were studied for their tertiary alkaloid contents to reveal the chemotypes of this species. Antimicrobial activities of methanol and alkaloid extracts of the samples were also carried out using a microbroth dilution technique against nine ATCC strains.

The structures of the alkaloids were elucidated through spectroscopic analysis and TLC by direct comparison with authentic samples. It has been shown that five samples contain only rhoeadine types, whereas two samples yielded rhoeadine type together with protopine. Existence of roemerine was shown in three samples (Table 4). These findings confirm the results of previous researchers on *P. rhoeas* which reported the presence of rhoeadine types in most of the samples investigated (Kalav and Sarıyar 1989; Ünsal et al. 2007).

On the basis of alkaloid yield, *P. rhoeas* samples from Turkey and Cyprus were found to be very similar. The yields of alkaloid extracts of Turkey samples were in the range of 0.07–0.19%, while Cyprus samples were in the range of 0.09–0.18%.

The results of the antimicrobial activity by the microbroth dilution method of *P. rhoeas* species are presented in Table 5. It could be observed that the alkaloid extracts from P8 and P9 and methanol extract from P5 showed a good antimicrobial activity against *S. aureus*, *S. epidermidis* and *K. pneumoniae*. No activity was seen against *P. mirabilis*. The most significant activity was observed with the alkaloid extract of P8 against *S. aureus* with a MIC value of 1.22 μg/mL. Alkaloid extracts of P8 and P9 were shown to be especially active against *S. aureus*, *S. epidermidis* and *K. pneumoniae* with MIC values ranging between 9.7 and 19 μg/mL. The antibacterial effect of these two extracts may be related to their major alkaloid roemerine. Also a moderate activity was obtained by the methanol extract of P5 against *S. aureus* and alkaloid extract of P8 against *K. pneumoniae* with a MIC value of 39 μg/mL.

**Table 5:** Minimum inhibitory concentrations (μg/mL) of extracts from *Papaver rhoeas*L.

Micro-organisms										
		Gram-positive bacteria			Gram-negative bacteria			Fungi		
Species/referencestandard	Extract	*Staphylococcus aureus*	*Staphylococcus epidermidis*	*Klebsiella pneumoniae*	*Proteus mirabilis*	*Escherichia coli*	*Pseudomonas aeruginosa*	*Candida albicans*	*Candida parapsilosis*	*Candida tropicalis*
P1	M	156	–	–	–	–	–	–		
	A	–	625	–	–	–	–	–		
P2	M	–	625	–	–	–	–	–		
	A	156	312	–	–	–	–	–		
P3	M	156	625	–	–	–	–	–		
	A	312	625	–	–	–	–	–		
P4	M	312	–	–	–	–	–	–		
	A	–	–	–	–	–	–	–		
P5	M	39	156	–	–	–	312	–		
	A	312	625	–	–	–	–	–		
P6	M	312	–	–	–	–	–	–		
	A	156	625	–	–	–	–	625		
P7	M	156	312	–	–	–	312	–		
	A	–	625	–	–	–	–	–		
P8	M	312	–	312	–	–	–	–		
	A	1.22	9.7	39	–	312	156	2.4		
P9	M	–	–	312	–	–	–	–		
	A	9.7	19	19	–	78	78	9.7		
P10	M	312	625	–	–	–	–	156		
	A	9.7	19.5	–	–	–	–	2.4		
P11	M	1250	–	–	–	–	–	312		
	A	156	625	–	–	–	–	312		
Roemerine								4.8	4.9	2.4
Mecambrine								39	39	9.8
Cefuroxime-Na		1.2	9.8	4.9	2.4	4.9				
Ceftazidime							2.4			
Clotrimazol								4.9		

M: methanol; A: alkaloidal; –: not active.

Only roemerine and mecambrine were tested against *Candida parapsilosis* and *Candida tropicalis*.

Comparison of antifungal activity of all the extracts tested, P8, P9 and P10 alkaloid extracts exhibited a remarkable activity against *Candida albicans* with MIC values of 2.4, 9.7 and 2.4 μg/mL, respectively.

Rhoeadine-type skeletons represent the main alkaloids of *P. rhoeas* of Turkish origin, except for the southeast region sample (P8), whereas aporphine-type alkaloids were found in the two native *P. rhoeas* species of northern Cyprus (P9, P10). The extracts containing rhoeadine type (P1, P2, P4, P6, P7) and rhoeadine–protopine type (P3, P5) alkaloids had no significant activity against the microorganisms tested except the activity of P5 against *S. aureus* with a MIC value of 39 μg/mL. The most potent extracts (P8, P9 and P10) have been found to contain roemerine as the major alkaloid which has an aporphine alkaloid skeleton and a methylenedioxy moiety. This alkaloid has been shown to possess a variety of pharmacological properties, such as vasodilator (Valiente et al. 2004), anthelmintic (Lin et al. 2014) and antiplasmodial (Baghdikian et al. 2013) activities. One of the derivatives of roemerine, (+)-roemerine MeI, was reported to have strong antibacterial activity against Gram-positive bacteria, including *Bacillus cereus*, *Micrococcus* sp. and *Staphylococcus aureus* (Tsai et al. 1989). Previous studies showed that roemerine had a certain activity against fungal pathogens such as *Candida albicans*, *Candida glabrata*, *Candida krusei*, *Candida parapsilosis* and *Cryptococcus neoformans* (Rao et al. 2009). Furthermore, Agnicorti et al. (2008) have reported that this alkaloid has significant antifungal activity against *Candida albicans*. Roemerine was found to have great bioavailability (84%) in Sprague–Dawley rats and it was suggested that roemerine could be taken orally instead of intravenously (Liu et al. 2014). Hence, roemerine could be a promising drug lead for the clinical treatment of fungus infections.
